# Disposable Face Masks for Noninvasive Drug Detection: A Proof-of-Concept Study with Cough Syrup Constituents

**DOI:** 10.1021/acs.analchem.5c01129

**Published:** 2025-05-05

**Authors:** Hei-Tak Tse, Jian Zhen Yu, Zongwei Cai, Wan Chan

**Affiliations:** † Department of Chemistry, 58207 The Hong Kong University of Science and Technology, Clear Water Bay, Kowloon 999077, Hong Kong; ‡ Eastern Institute of Technology Ningbo, Ningbo, Zhejiang 315200, China

## Abstract

Current drug detection methods, such as blood and urine analysis, are often invasive and raise ethical and privacy concerns. This study demonstrates that breathing through typical polypropylene-based meltblown cloth face masks is an efficient and user-friendly method for collecting drugs from exhaled breath for analysis. By using codeine, ephedrine, guaifenesin, and chlorpheniramine found in cough syrup as model compounds, we found that these face masks achieved a collection efficiency exceeding 92% for the tested drugs. The analysis yielded pharmacokinetic parameters—such as half-life (*t*_1/2_), time to maximum concentration (*T*_max_), and detection window—that were comparable to those obtained through parallel urine analysis. Given the increasing demand for noninvasive drug detection methods due to the rising abuse of substances like marijuana and fentanyl, this method is expected to have broad applications in forensic analysis and drug development.

## Graphical abstract

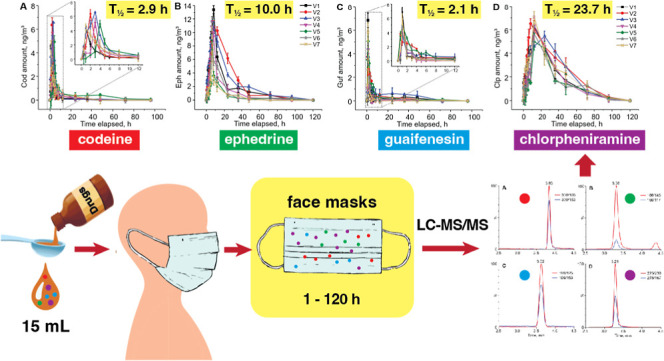

## Introduction

Drug abuse is associated with a wide range of adverse consequences, including social, legal, economic, and health-related issues.^1–3^ Accurate identification of individuals who engage in illicit drug use is essential for both forensic and medical applications.^4–6^ Current methods for detecting drug abuse utilize various biological matrices, including urine, blood, breath, saliva, sweat, and hair analysis.^4,7–11^ Among these, urine testing remains the most commonly employed method due to its noninvasive nature, rapid processing time, and ability to detect a broad spectrum of drugs.^4,7,12^ However, urine analysis can be influenced by factors such as hydration status, which may affect test results, and it raises privacy concerns, particularly during supervised sampling procedures.^13,14^

Nevertheless, the noninvasive detection of drugs and their metabolites is of significant interest within forensic science, particularly given the increasing legalization of marijuana in various jurisdictions across Southeast Asia, the United States, and the European Union, as well as the alarming rise in fentanyl abuse in the USA.^15,16^ Furthermore, the ability to noninvasively detect and monitor pharmaceuticals presents a promising approach for improving patient monitoring and adherence to treatment regimens. This method could potentially eliminate the need for routine blood sampling, which is invasive, costly, and often requires ethical approval.^17,18^

Among the noninvasive drug testing methods, exhaled breath analysis is emerging as a novel avenue for drug detection, as it contains bioaerosols that carry nonvolatile substances, such as drugs and their metabolites, expelled from the body.^19–22^ To facilitate this process, sampling devices equipped with microparticle filters have been developed and tested for trapping drugs present in the exhaled breath of patients.^8,23,24^ However, these methods currently face challenges related to low detection rates and require improvements in analytical sensitivity.^4^ It is likely that the high linear velocity of exhaled breath passing through the sampling device negatively impacts the efficiency of drug collection, potentially compromising the sensitivity of detection.^4,23^

Surgical face masks, known for their high bioaerosol filtration efficiency,^25^ have been extensively utilized to protect individuals from airborne pathogens and carcinogens, including the severe acute respiratory syndrome coronavirus 2 (SARS-CoV-2) and polycyclic aromatic hydrocarbons,^26–29^ respectively. Additionally, research has demonstrated that surgical masks are highly effective in trapping antimicrobial resistance gene-bearing pathogens in the exhaled breath of patients suffering from chronic obstructive pulmonary disease.^30,31^ Although quantitative analysis is still pending, Huang et al. reported using face masks to collect and detect ingested acetaminophen in exhaled breath.^32^ Therefore, we propose that face masks can effectively trap drugs in exhaled breath and serve as a convenient air sampler for the detection of drugs of abuse.^33–36^

We initiated the study by assessing the efficiency of fabric masks in trapping codeine (Cod; Table 1), ephedrine (Eph), guaifenesin (Guf), and chlorpheniramine (Clp) in exhaled breath. These compounds are active pharmaceutical ingredients commonly found in cough syrup formulations (Table 1). The results provided a critical evaluation of analyte loss during air sample collection, facilitating necessary corrections to improve the detection accuracy. Next, we investigated the dose-dependent accumulation of these compounds by instructing three volunteers to wear surgical masks after consuming varying quantities of cough syrup.

**Table 1:**
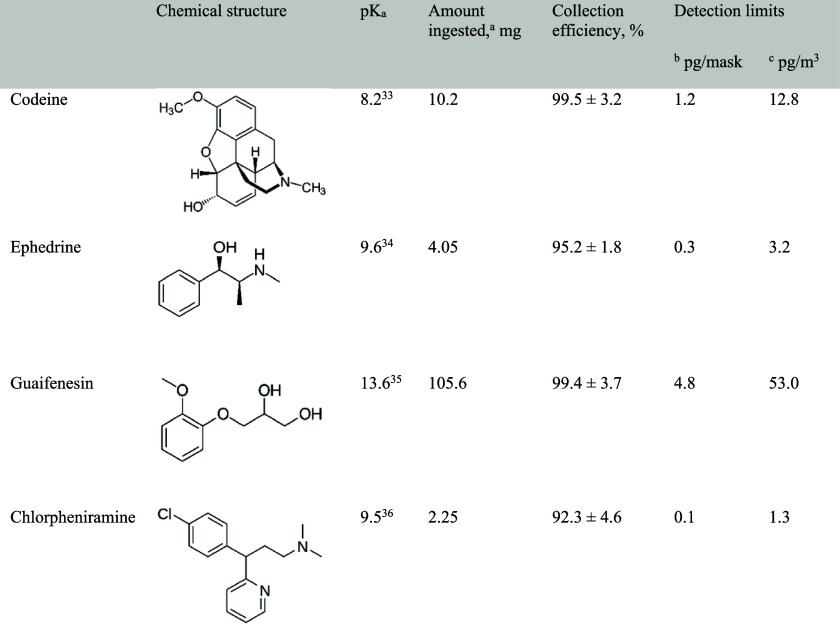
Summary of Active Ingredients in Cough Syrup, Collection Efficiencies, and Detection Limits

a Calculated based on: ingesting 15 mL cough syrup.

b One-half of a mask used in each analysis.

c The volume of air breathed in 15 min.

Additionally, we monitored the concentration profiles of these drugs in the exhaled breath of seven volunteers over an extended period of 120 h following a single oral dose of cough syrup. Using liquid chromatography-tandem mass spectrometry (LC–MS/MS) combined with stable-isotope dilution, our methodology demonstrated high accuracy and precision in drug monitoring, successfully detecting individual drugs up to 4 days postingestion. Finally, we compared the analytical results from breath analysis to those from urine analysis. Our findings indicated that drug detection in exhaled breath provided a longer detection window compared to urine analysis, suggesting that breath analysis may serve as a viable alternative for identifying drug abuse.

## Materials and Methods

### Caution

Codeine-containing cough syrup may be addictive and should only be administered under medical supervision. Fabric masks utilized for drug sampling may harbor pathogenic microorganisms and should be handled with appropriate personal protective equipment within biosafety cabinets. It is imperative that these masks are sterilized on both sides using 75% ethanol immediately after use and stored in screw-capped glass tubes in a −20 °C refrigerator until processed for analysis. Furthermore, the use of fabric masks as a sampling or testing method is not recommended during pandemic situations or in areas experiencing outbreaks.

### Chemicals and Reagents

All chemicals and reagents utilized in this study were of the highest purity available and were used without further purification. Codeine, ephedrine, guaifenesin, chlorpheniramine, creatinine, codeine-*d*_6_ (Cod-*d*_6_), chlorpheniramine-*d*_6_ (Clp-*d*_6_), and creatinine-*d*_3_ were procured from Sigma-Aldrich (St. Louis, MO). Polypropylene-based face masks (Haishi Hainuo; Qingdao Hainuo Biological Engineering Co., Ltd.) with a bacterial filtration efficiency (BFE) of ≥98% were purchased from a local pharmacy in Hong Kong, along with cough syrup containing codeine, ephedrine, guaifenesin, and chlorpheniramine as active ingredients (Table S1). HPLC-grade methanol and acetonitrile were obtained from Tedia (Fairfield, OH). Deionized water was purified by using a Pall Cascada laboratory water purification system (Port Washington, NY) and was used in all experiments.

### Sample Collection

The collection efficiency of fabric masks for the aforementioned drugs in cough syrup was tested as previously reported,^28,29^ with modifications to suit the analysis of drugs in exhaled breath. In brief, three volunteers consumed 15 mL of cough syrup and were instructed to clean their lips with clean tissue paper. One hour postconsumption, the volunteers were directed to wear double-layered masks prepared by stapling together two identical masks for 15 min, during which time they were instructed to avoid talking, coughing, or engaging in vigorous exercise. The mask layers were subsequently processed and tested separately, and the collection efficiency was calculated using eq 1

1 $$collection\;efficiency=\frac{amount\;of\;drug\;in\;inner\;mask}{total\;amount\;of\;drug\;in\;the\;two\;mask\;layers}\times 100\%$$

The dose-dependent accumulation of the respective drugs on fabric masks was assessed similarly, employing single masks with a BFE of ≥98% for 15 min at 1 h after taking 5 mL, 10 mL, or 15 mL of cough syrup. Each dose administration was separated by a minimum interval of 1 week.

Similarly, the concentrations of drugs in exhaled breath were measured at various time points following drug administration in seven individuals, each of whom received a single oral dose of 15 mL of cough syrup. At 0.25, 0.5, 1, 2, 3, 4, 5, 6, 8, 12, 24, 36, 48, 72, 96, and 120 h postadministration, the volunteers were instructed to wear a new mask for 15 min. After sample collection, the masks were immediately sterilized and stored at −20 °C until processed for analysis (Figure S1).

### Sample Preparation

Prior to analysis, each mask was divided into two equal halves. One-half was returned to screw-capped glass tubes and stored at −20 °C for potential future analysis. A 10 μL aliquot of an internal standard mixture (Cod-*d*_6_ and Clp-*d*_6_ at 10 ng/mL in methanol) was added dropwise to the other half. This half was then cut into 1 cm × 1 cm pieces and soaked in 10 mL of methanol in a screw-capped glass tube, followed by sonication at room temperature for 30 min. Five milliliters of the extraction solution were transferred to another glass tube and dried under a nitrogen stream, and the resulting residue was redissolved in 0.1 mL of methanol before being transferred to an HPLC vial for LC–MS/MS analysis.

### LC–MS/MS Analysis

LC–MS/MS analysis of Cod, Eph, Guf, and Clp was conducted by using an Acquity UPLC system coupled with a Waters TQ-XS triple quadrupole LC–MS/MS system (Waters Corporation; Milford, MA). Ten microliters of the sample extracts were injected into a Luna C18 column (100 mm × 2.0 mm i.d., 3 μm; Phenomenex; Torrance, CA). The column was eluted at a constant flow rate of 300 μL/min, beginning with a mobile phase composition of 90% A (10 mM ammonium acetate in water) and 10% B (acetonitrile) held for 1 min. A linear gradient was then applied, increasing to 40% B over 3 min, ramping to 75% B over 3 min, and further increasing to 100% B over 1 min, followed by a hold at 100% B for an additional 3 min before re-equilibration.

The LC eluate from the third to the seventh minutes was directed to the LC–MS/MS system for the drug analysis. The mass spectrometer operated in multiple reaction monitoring (MRM) mode, utilizing the following MRM transitions for quantitative analysis: *m*/*z* 300/165 for Cod, *m*/*z* 166/148 for Eph, *m*/*z* 199/125 for Guf, *m*/*z* 275/230 for Clp, *m*/*z* 306/165 for Cod-*d*_6_, and *m*/*z* 281/230 for Clp-*d*_6_. Qualitative analysis of Cod, Eph, Guf, and Clp was conducted using MRM transitions at *m*/*z* 300/153, *m*/*z* 166/117, *m*/*z* 199/163, and *m*/*z* 275/167, respectively.

### Calibration Curve, Method Validation, and Quality Control

Calibration curves for the quantitative analysis of Cod, Eph, and Guf were constructed by plotting the peak area ratios of these compounds to Cod-*d*_6_ against their respective concentrations in the working standards. The calibration curve for Clp was established by plotting the peak area ratios of Clp to Clp-*d*_6_ versus the amount of Clp in the working standards (Figure S2). The extraction efficiency of drugs from masks was evaluated by spiking various amounts of drugs into drug-free masks, followed by extraction and analysis of the recovered drug amounts using LC–MS/MS (Table S2). Intraday and interday precisions of the method were assessed by analyzing samples separately on the same day (*n* = 7) and over a period of 7 days, respectively. Reagent and method blanks were included in every batch of 20 samples, in accordance with EPA QA/G-5 guidelines.^37^

### Tidal Volume and Breathing Rate Measurement

Tidal volumes of the seven volunteers (male and female, ages 22 to 32) were estimated using a spirometer, as described previously.^28,30^ The tidal volume for individual volunteers while wearing masks was measured at 0.3 ± 0.1 L/breath. Breathing rates were calculated by counting and averaging breaths over a 5 min interval, resulting in a measurement of 16.0 ± 5.4 breaths/min. This rate is equivalent to sampling air at a rate of 4.8 L/min (Table S3). The total volume of air drawn through the masks was calculated by multiplying the average breathing rate of the volunteers using eq 2

2 totalsamplingvolume=tidalvolume×breathingrate×samplingtime

### Urine Sample Analysis

In parallel with the collection of drugs in exhaled breath using the mask-based sampling method, urine samples from volunteers who ingested 15 mL of cough syrup were also collected within the similar time frame and stored at −20 °C for comparative analysis. Before analysis, the urine samples were thawed at room temperature and centrifuged at 6,000*g* to remove coarse material. Subsequently, 1 mL of the supernatant was combined with 10 μL of the internal standard mixture (containing 10 ng/mL Cod-*d*_6_ and Clp-*d*_6_) and loaded onto C18 solid-phase extraction (SPE) columns for sample cleanup, following previously established protocols.^38^ The SPE eluate was then dried under a nitrogen stream and redissolved in 100 μL of methanol before analysis using the same LC–MS/MS method described earlier for mask sample analysis. In a separate LC–MS/MS analysis, urinary creatinine concentration was determined using creatinine-*d*_3_ as an internal standard, as reported previously.^39^

### Statistical Analysis

Detection limits were calculated as the amount of drug (in half a mask) that generated an analytical signal three times the noise level, as described previously.^28^ Grubb’s test was conducted to identify outliers. The biological half-life (*t*_1/2_) of individual drugs was calculated as the time for their concentration in exhaled breath or urine to decrease from its maximum (*C*_max_) to half of *C*_max_. The elimination rate constant (*k*_el_) and mean residence time (MRT) of each drug were determined from the slope of the elimination phase of the exhaled breath or urine concentration curve plotted on a semilogarithmic scale and by taking the reciprocal of the elimination rate constant, respectively. The Pearson correlation coefficient was calculated to examine the linear relationship between exhaled concentration and urine concentration, with the level of significance set to 0.05.

## Results and Discussion

### Characterization of Collection Efficiencies

The collection efficiencies of surgical face masks for target drugs were characterized as previously reported,^28,29^ with modifications. In brief, three volunteers consumed 15 mL of cough syrup and were instructed to wear double-layered masks for 15 min, starting one hour after drug administration. Here, it is worth mentioning that the sampling was conducted at one hour after the drug administration because results of preliminary study showed most of the four drugs in exhaled breath reached maximum concentration at around 1 h postadministration. The 15 min sampling duration was optimal, providing a signal intensity that was not excessively high for samples with high drug concentrations while remaining sufficiently strong for quantitative analysis in samples with low drug concentrations.

Using the LC–MS/MS method outlined in the Materials and Methods section, the amount of drugs trapped on each mask was subsequently quantified. Collection efficiencies were calculated using eq 1, where the amounts of a drug collected on the inner mask were divided by the total amount collected across both the inner and outer masks to determine the trapping efficiency. Figure 1 shows typical chromatograms obtained from LC–MS/MS analysis of Cod, Eph, Guf, and Clp, in masks worn by one of the volunteers.

**Figure 1:**
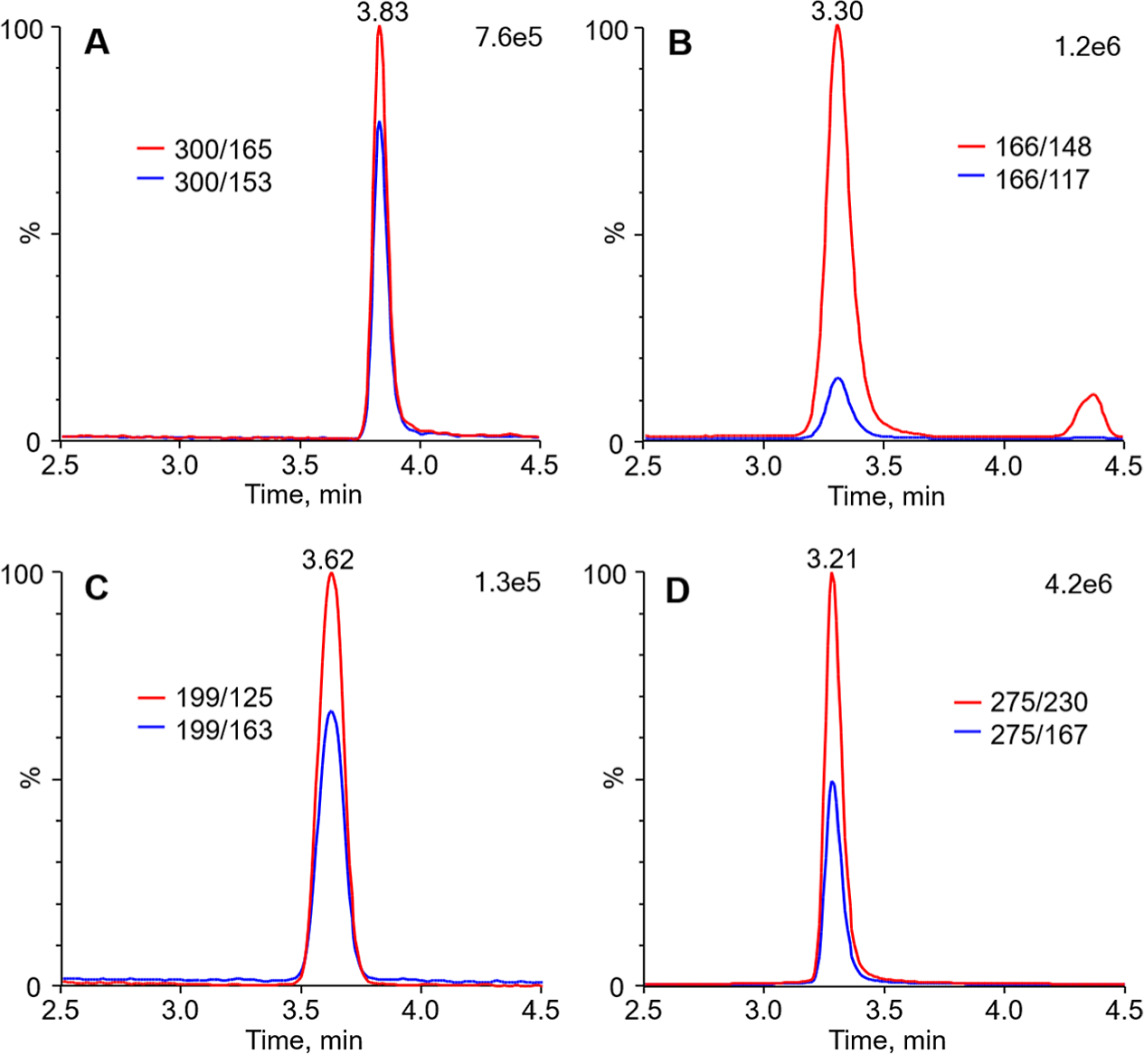
Reconstructed chromatograms from LC–MS/MS analysis of codeine (A), ephedrine (B), guaifenesin (C), and chlorpheniramine (D) in a mask worn by one of the volunteers for 15 min at 1 h after consuming 15 mL of cough syrup.

The analysis revealed that the majority of the drugs present in exhaled breath were effectively captured on the inner mask with collection efficiencies exceeding 92% for all target compounds (Table 1). Consequently, single-layered face masks were utilized as-is in subsequent studies. Considering the distinct chemical properties of the four representative drugs (Table 1), such as Cod, an opioid closely related to morphine, these findings demonstrate that breathing through face masks offers a quantitative method for detecting drugs in exhaled breath, thereby supporting drug abuse testing and facilitating the assessment of absorption, distribution, metabolism, and excretion (ADME) in drug development.

### Dose Dependence of Drug Accumulation in Masks

Following the demonstration that face masks are highly effective in trapping drugs in exhaled breath, we proceeded to investigate the dose-dependent accumulation of four drugs on the masks used through LC–MS/MS analysis. Volunteers consumed 5, 10, or 15 mL of cough syrup and were instructed to wear a mask for 15 min, starting one hour after drug administration. The amount of each drug trapped on the masks was then quantified, and the concentrations of drugs in the exhaled breath of the three volunteers, normalized to breathing volume, are presented in Figure 2.

**Figure 2:**
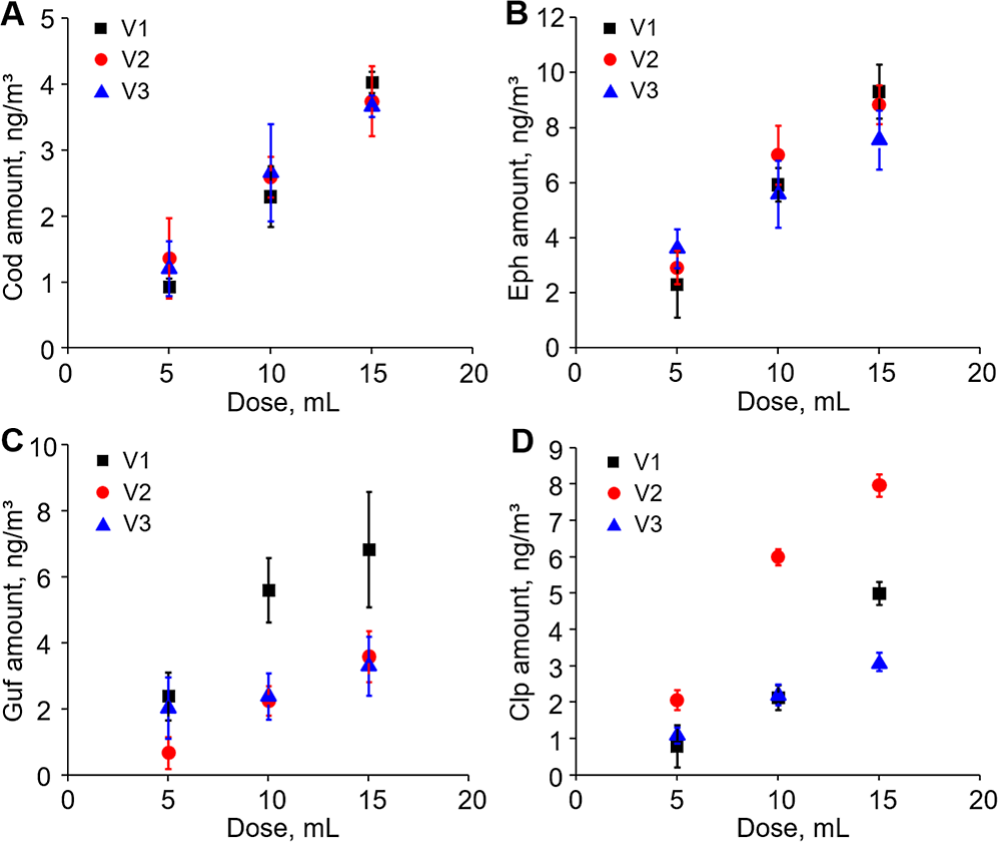
Concentrations of codeine (A), ephedrine (B), guaifenesin (C), and chlorpheniramine (D) in exhaled breath of three volunteers at 1 h after consuming different amounts of cough syrup, as determined by dividing the total amounts of individual drugs trapped on the after-use masks by the breathing volume in the 15 min of mask wearing. The data represent means ± SD for three independent experiments.

A clear dose-dependent accumulation of the four drugs was observed on the masks worn by all three volunteers, with a linear regression coefficient (*r* ^2^) exceeding 0.94. These results indicate that the collection of drugs in exhaled breath using face masks is quantitative, with the amount of drugs trapped on the masks closely correlating with the amount of drug taken by the volunteers. This suggests that breathing through face masks may serve as an efficient and user-friendly method for identifying drug abusers.

Interestingly, Guf, despite being the most concentrated drug among the four active ingredients in cough syrup (Table 1), with a concentration over ten times higher than the others, was found to be present at only a slightly higher concentration than the other three drugs in volunteer 1. In contrast, its concentrations were lower than those of the other drugs in the exhaled breath of volunteers 2 and 3. This observed difference may be attributed to Guf’s rapid metabolism, which facilitate its elimination from the body.^40^ Specifically, Guf is rapidly metabolized in the liver via both oxidation and demethylation, with most of it being processed within a few hours after the administration.^40^ Consequently, lower than expected concentrations of Guf were detected in the exhaled breath of volunteers.

### Drug Clearance Study by Breathing through Face Masks

We then investigated the potential of using breathing through face masks as a sampling method to study *t*_1/2_ of the aforementioned drugs in humans. Seven volunteers were recruited and administered 15 mL of cough syrup. The concentrations of the drugs in exhaled breath were determined by having the volunteers wear and breathe through a face mask for 15 min at various time points after drug administration. The amounts of drugs trapped on the masks were quantified using LC–MS/MS analysis.

The analysis revealed *C*_max_ in exhaled breath at 1.9 ± 1.0 h, 6.9 ± 1.1 h, 0.9 ± 0.2 h, and 13.1 ± 5.0 h after drug administration for Cod, Eph, Guf, and Clp, respectively (Figure 3). All drugs remained detectable at ng/m^3^ levels for over 2 days following administration, with Eph exhibiting the highest concentration, reaching a *C*_max_ of 10.8 ± 1.6 ng/m^3^.

**Figure 3:**
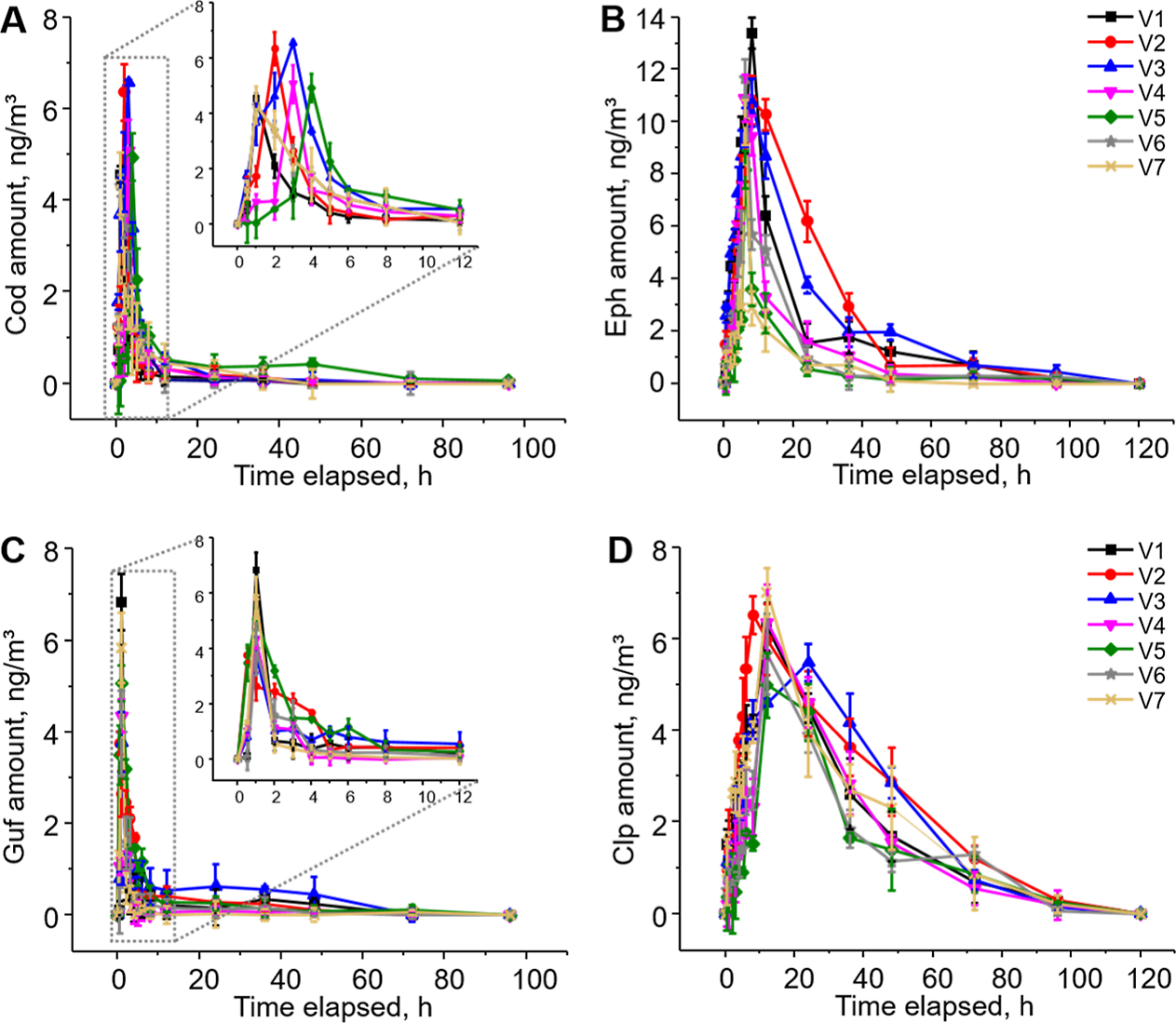
Concentrations of codeine (A), ephedrine (B), guaifenesin (C), and chlorpheniramine (D) in the exhaled breath of seven volunteers at various time points after consuming 15 mL of cough syrup. Concentrations were determined by dividing the total amounts of drugs trapped on the used masks by the breathing volume during the 15 min of mask wearing. Data represent means ± SD from three independent experiments.

The differentiated *T*_max_ values for the four targeted drugs provide additional evidence, confirming the origin of the drugs on the mask. Specifically, the *C*_max_ values observed from 1 to 12 h postadministration, particularly after the third sampling point for Guf and the tenth sampling point for Clp, support the conclusion that the drugs detected on the mask originated from exhaled breath rather than saliva. If the drugs had come from saliva, the highest concentrations would have been detected immediately after drug administration at the first sampling point.

The *t*_1_/_2_ values for the drugs, defined as the time taken for their concentrations to decrease from *C*_max_ to half of *C*_max_ in exhaled breath, were found to be 2.9 ± 0.8 h for Cod, 10.0 ± 2.8 h for Eph, 2.1 ± 0.9 h for Guf, and 23.7 ± 3.2 h for Clp (Table 2). These results are consistent with those reported in the literature; for instance, the plasma t_1_/_2_ for Cod is cited as 2–3 h,^41,42^ while those for Eph, Guf, and Clp are approximately 6 h,^43^ 1 h,^40^ and 20 h,^44^ respectively, although significant interpatient variability has been observed.

**Table 2:** Summary of Pharmacokinetic Parameters for Drugs in Cough Syrup in Exhaled Breath Captured on Masks by the Mask Wearing Method and in Urine

exhaled breath analysis					
	*T*_max_, h	*t*_1/2_, h	*k*_el_, h^–1^	MRT, h	detection window, h
codeine	1.9 ± 1.0	2.9 ± 0.8	0.3 ± 0.1	3.9 ± 0.8	61.3 ± 12.7
ephedrine	6.9 ± 1.1	10.0 ± 2.8	0.1 ± 0.04	14.5 ± 4.1	92.6 ± 9.1
guaifenesin	0.9 ± 0.2	2.1 ± 0.9	0.4 ± 0.3	4.8 ± 4.2	65.1 ± 18.1
chlorpheniramine	13.1 ± 5.0	23.7 ± 3.2	0.03 ± 0.01	34.2 ± 4.6	99.4 ± 9.1

urine analysis					
	*T*_max_, h	*t*_1/2_, h	*k*_el_, h^–1^	MRT, h	detection window, h
codeine	2.1 ± 1.3	3.3 ± 0.7	0.3 ± 0.1	4.6 ± 2.1	68.2 ± 8.3
ephedrine	2.7 ± 1.8	6.2 ± 0.6	0.1 ± 0.02	10.4 ± 3.3	79.9 ± 11.4
guaifenesin	1.0 ± 0.7	1.8 ± 0.9	0.4 ± 0.3	3.5 ± 2.4	57.6 ± 3.2
chlorpheniramine	7.1 ± 3.9	19.2 ± 14.8	0.1 ± 0.04	17.3 ± 4.2	90.9 ± 7.9

### Comparative Urine Analysis

To critically evaluate the performance of the developed face mask-based sampling device for detecting drugs in exhaled breath, we also collected and analyzed urine samples from the seven volunteers at time points similar to those for the breath analysis (Figure 4). Similar urinary concentration profiles were observed for the drugs compared to those in breath analysis.

**Figure 4:**
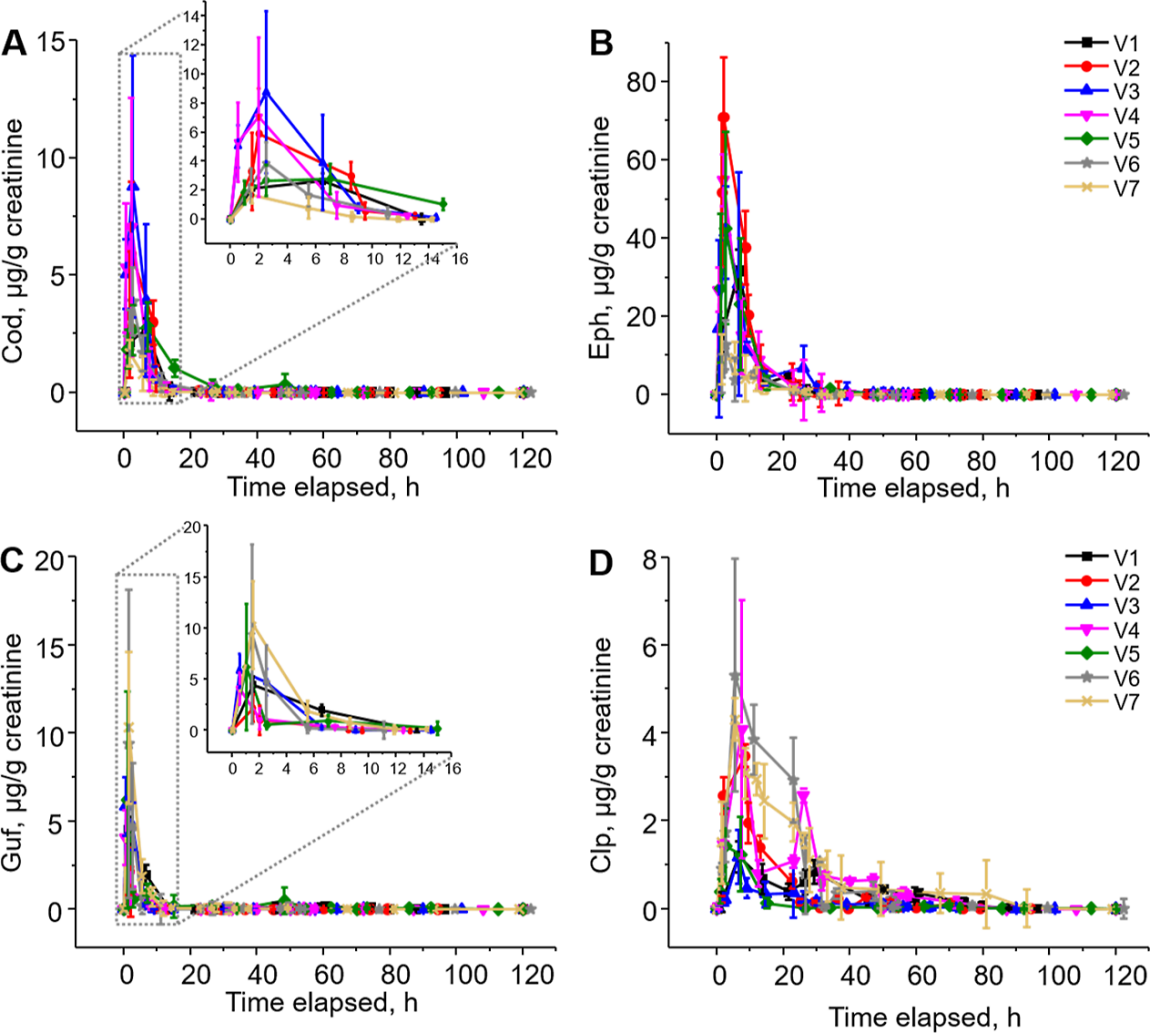
Urinary concentrations of codeine (A), ephedrine (B), guaifenesin (C), and chlorpheniramine (D) in the urine of seven volunteers at various time points following the ingestion of 15 mL of cough syrup. Concentrations are normalized to creatinine levels. Data are presented as means ± SD from three independent experiments.

Subsequent correlation analysis revealed a strong linear relationship between exhaled concentrations and urine concentrations for Cod and Eph, with Pearson correlation coefficients (*r*) exceeding 0.91 (Figure 5). Additionally, the detection windows and half-lives (*t*_1/2_) in urine were consistent with those observed in the breath analysis (Table 2). These findings suggest that breathing through face masks is a noninvasive and user-friendly method for identifying drug abuse and may also be useful for assessing drug absorption.

**Figure 5:**
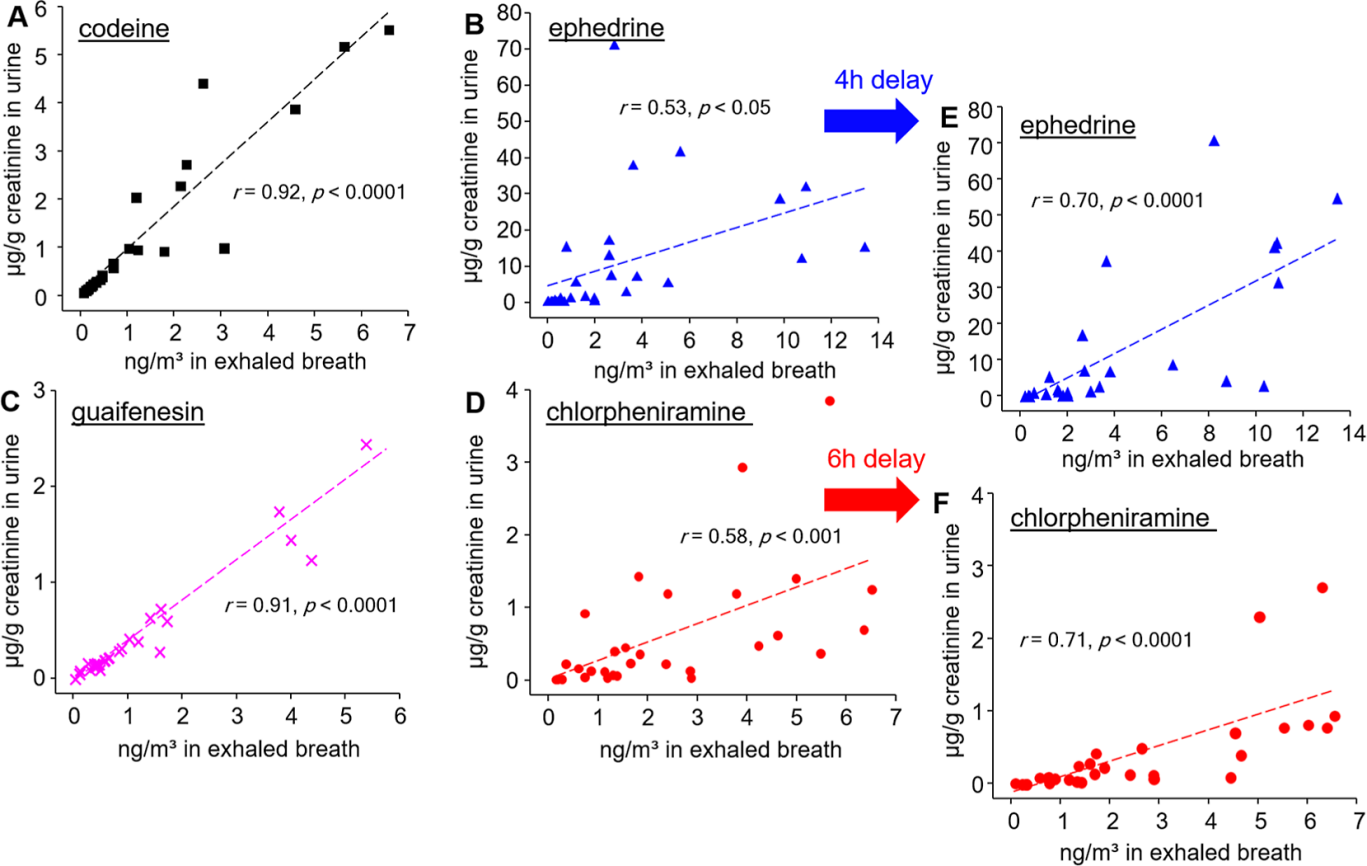
Correlations of codeine (A), ephedrine (B), guaifenesin (C), and chlorpheniramine (D) concentrations in exhaled breath and urine after ingesting 15 mL of cough syrup by seven volunteers. Shown in panels E and F are the correlation of time-delayed breath and urinary concentrations of ephedrine and chlorpheniramine, respectively. Urinary drug concentrations are normalized to creatinine levels. The insets show the statistical results of Pearson’s regression analysis.

Here, it is important to note that the data for Eph and Clp from the mask and urine analyses did not correlate well, with Pearson correlation coefficients of r = 0.53 for Eph and r = 0.58 for Clp. This discrepancy can be attributed to the different distribution of the two drugs in the lung and kidney and, as a result, distorted concentration profiles observed in exhaled breath and urine, as indicated by their varying *T*_max_ values. For example, Eph had a *T*_max_ of 6.9 ± 1.1 h in breath compared to 2.7 ± 1.8 h in urine, while Clp had *T*_max_ values of 13.1 ± 5.0 h in breath versus 7.1 ± 3.9 h in urine (see Table 2). The delayed *T*_max_ is likely due to both drugs being weak bases (p*K*_a_ ∼ 9.5; Table 1). At physiologically relevant pH (e.g., 7.4), they predominantly exist in an ionized form, which facilitates their solubility in blood and renal clearance.^45–47^ Furthermore, this also results in low volatility, making them eliminated slower in exhaled breath. Consequently, the shorter *T*_max_ for Clp and Eph was observed in urine compared with exhaled breath.

Despite this, the delayed *T*_max_ for Eph and Clp in exhaled breath allows for a longer drug detection window compared to that in urine analysis. Specifically, the detection windows for Eph and Clp in exhaled breath were 92.6 ± 9.1 h and 99.4 ± 9.1 h, respectively, while the detection windows in urine were 79.9 ± 11.4 h and 90.9 ± 7.9 h, respectively. A time-delay correlation analysis, with urinary concentrations of Eph and Clp delayed by 4 and 6 h, respectively, showed a reasonable agreement with the exhaled breath analysis, yielding correlation coefficients of r = 0.70 for Eph and r = 0.71 for Clp (Figure 5).

### Assessment of Method Greenness and Practicality

The greenness of the analytical procedures for the developed method was evaluated based on their environmental friendliness and safety for humans, using the Analytical Greenness Calculator (AGREE)^48^ and the modified Green Analytical Procedure Index (MoGAPI).^49^ The results are presented in Figure S3 of the Supporting Information. Overall, both tools indicated that our method, from sample collection to instrumental analysis, is environmentally friendly with AGREE and MoGAPI scores of 0.74 and 76, respectively, highlighting its sustainability.

Furthermore, we assessed the practical applicability of the method using the Click Analytical Chemistry Index (CACI),^50^ which yielded a score of 68 (Figure S3), suggesting that the method is practically acceptable for its intended applications.

## Conclusions

In this study, we evaluated the efficacy of using codeine, ephedrine, guaifenesin, and chlorpheniramine, derived from cough syrup, as model compounds to demonstrate that exhalation through polypropylene-based meltblown face masks represents a novel methodology for collecting drugs in exhaled breath for subsequent analytical assessment. Comparative analysis indicated that this method yielded pharmacokinetic parameters—such as half-life (*t*_1/2_), time to maximum concentration (*T*_max_), and detection window—comparable to those obtained through conventional urine analysis. We also assessed the greenness and practicality of our method using several metrics, which demonstrated that it is both environmentally friendly and practically acceptable. Given the distinct chemical properties of the four tested drugs, including codeine (Cod), an opioid closely related to morphine, as well as the noninvasive and user-friendly nature of this mask-based approach, we propose that this methodology has significant potential for identifying drug abuse and facilitating drug development processes.

## Supplementary Material



## Notes

Information of the cough syrup; efficiency and precision of extracting codeine, ephedrine, guaifenesin, and chlorpheniramine from mask samples for analysis; tidal volume, breath rate, and total sampling volume of volunteers; stability of drugs on masks upon storing at −20 °C; calibration curves of codeine, ephedrine, guaifenesin, and chlorpheniramine by the developed LC–MS/MS method; and assessment scores of AGREE, CACI, and MoGAPI for the developed method (PDF)
