# Supplementary material for: Disposable Face Masks for Noninvasive Drug Detection: A Proof-of-Concept Study with Cough Syrup Constituents
Source: Anal Chem. 2025 May 5;97(19):10424–32. doi: 10.1021/acs.analchem.5c01129 (PMC12096346; doi:10.1021/acs.analchem.5c01129)
Supplement: Supplementary file 1 [file ac5c01129_si_001.pdf]

SUPPORTING INFORMATION

Disposable Face Masks for Non-Invasive Drug Detection: A Proof-of-Concept Study with Cough Syrup Constituents

Hei-Tak Tse, [a] Jian Zhen Yu, [a] Zongwei Cai, [b] and Wan Chan [a,*]

[a] Department of Chemistry, The Hong Kong University of Science and Technology, Clear Water Bay, Kowloon 999077, Hong Kong.

[b] Eastern Institute of Technology Ningbo, Ningbo, Zhejiang 315200, China

E-mail: chanwan@ust.hk; Phone: +852 2358-7370; Fax: +852 2358-1594.

TABLE OF CONTENTS

Table S1. Information of the cough syrup used in the study. (*Page S3*)

Table S2. Efficiency and precision of extracting drugs in mask samples. (*Page S4*)

Table S3. Tidal volume, breath rate, and total sampling volume of volunteers participated in this study. (*Page S5*)

Figure S1. Relative abundance of drugs upon spiking to masks and storing at -20 °C. (*Page S6*)

Figure S2. Calibration curves used for quantitative analysis of Cod, Eph, Guf, and Clp. (*Page S7*)

Figure S3. Assessment scores of AGREE, CACI, and MoGAPI for the developed face mask-based sampling method for drug analysis. (*Page S8*)

Table S1. Information of the Cough Syrup used in this Study.

**Brand**	Madame Pearl's Cough Syrup
**Manufacturer**	Luxembourg Medicine Co. Ltd
**Origin**	Hong Kong
**Recommended dosage**	One tablespoonful (15mL) 3 times daily and two tablespoonfuls (30mL) at bedtime for adult
**Labelled concentration;** w/v, %	
Codeine Phosphate	0.090
Ephedrine HCl	0.033
Guaiphenesin	0.704
Chlorpheniramine Maleate	0.0210

Table S2. Efficiency and Precision of Extracting Codeine, Ephedrine, Guaifenesin, and Chlorpheniramine in Mask Samples.

	Accuracy			Precision	
	Spiked, ng/mask	Found, ng/mask [a]	Recovery, %	Interday, %RSD [a]	Intraday, %RSD [a]
Cod	0.01	0.01 ± 0.002	98.3	7.0	6.4
	0.1	0.10± 0.01	95.7	9.3	6.6
	1	1.01 ± 0.08	101.1	6.1	8.0
Eph	0.01	0.009 ± 0.001	88.4	5.7	6.4
	0.1	0.09 ± 0.01	86.4	3.1	8.1
	1	0.97 ± 0.04	97.3	6.3	4.2
Guf	0.01	0.009 ± 0.003	90.8	5.6	5.8
	0.1	0.09 ± 0.01	91.5	8.1	9.3
	1	0.88 ± 0.07	88.1	7.1	7.1
Clp	0.01	0.01 ± 0.004	98.9	10.2	4.5
	0.1	0.09 ± 0.01	91.0	6.3	4.5
	1	0.97 ± 0.06	97.1	4.1	5.7

[a] $n = 7$

Table S3. Tidal Volume, Breath Rate, and Total Sampling Volume of Volunteers Participated in this Study.

	V1	V2	V3	V4	V5	V6	V7
Breathing rate, breath/min	20.7 ± 2.3	9.7 ± 1.2	21.3 ± 2.5	13.3 ± 0.6	22.7 ± 1.2	15.0 ± 1.0	9.7 ± 0.6
Tidal volume, L/breath	0.17 ± 0.02	0.35 ± 0.03	0.26 ± 0.02	0.25 ± 0.02	0.15 ± 0.003	0.5 ± 0.04	0.5 ± 0.02
Total sampling volume, m^3	0.05 ± 0.01	0.05 ± 0.002	0.08 ± 0.002	0.05 ± 0.003	0.05 ± 0.003	0.11 ± 0.02	0.07 ± 0.002

* The data represent means ± SD for three independent experiments.

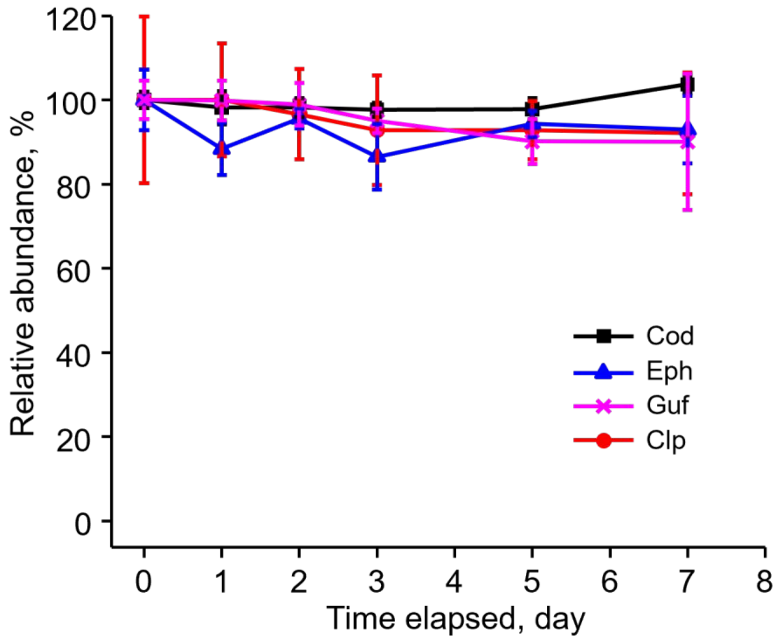

Figure S1. Relative abundance of drugs upon spiking to masks and storing at -20 °C for up to 7 days. Data represent mean value ± SD from three independent experiments.

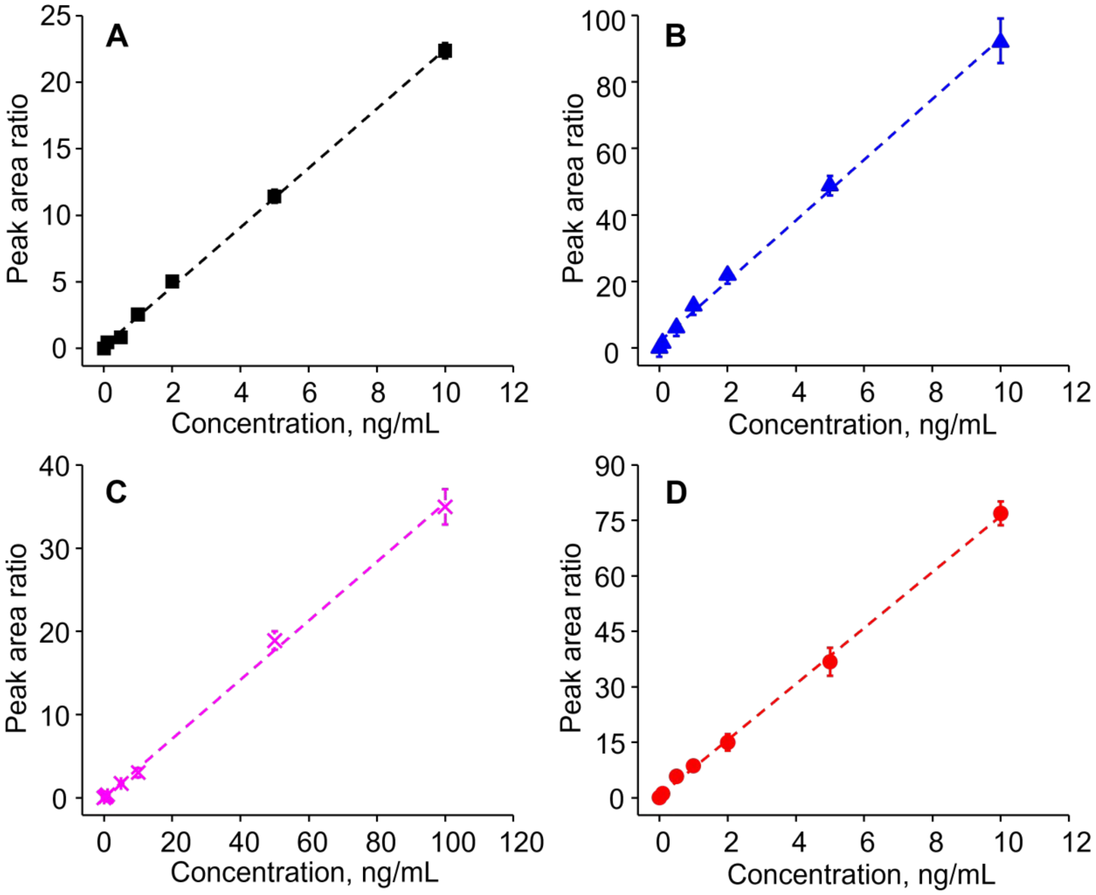

Figure S2. Calibration curves used for quantitative analysis of codeine (A), ephedrine (B), guaifenesin (C), and chlorpheniramine (D). Fitting the data by linear regression yielded lines with the following equations: Cod (A): y = 2.23x + 0.15 (r^2 = 0.99); Eph (B): y = 9.11x + 1.89 (r^2 = 0.99); Guf (C): y = 0.35x + 0.01 (r^2 = 0.99); Clp (D): y = 7.56x + 0.52 (r^2 = 0.99). Data represent mean value ± SD from three independent experiments.

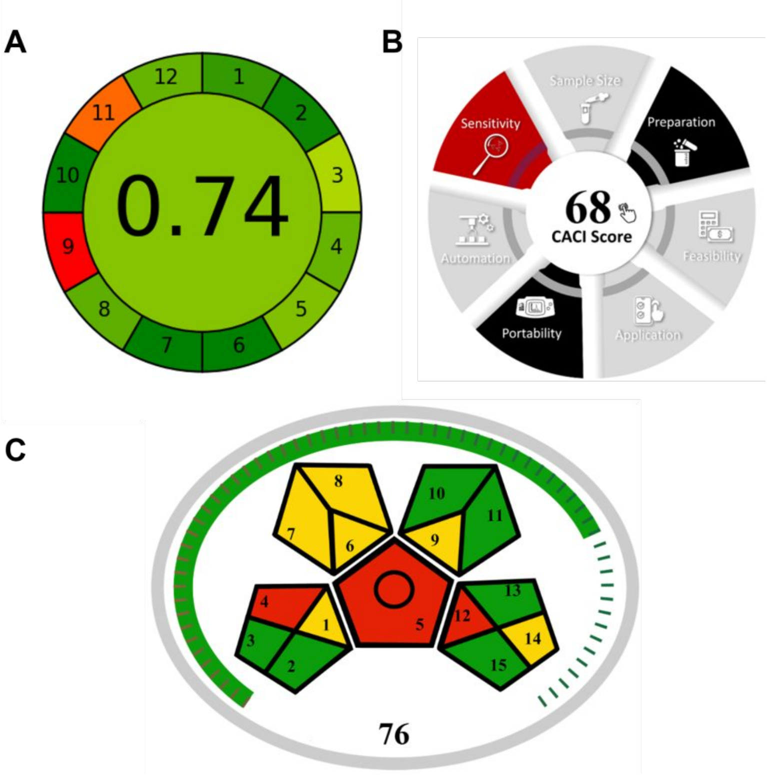

Figure S3. Assessment score of AGREE (A), CACI (B), and MoGAPI (C) for the developed face mask-based sampling method for drug analysis.
